# Barriers to Effective Municipal Solid Waste Management in a Rapidly Urbanizing Area in Thailand

**DOI:** 10.3390/ijerph14091013

**Published:** 2017-09-04

**Authors:** Nachalida Yukalang, Beverley Clarke, Kirstin Ross

**Affiliations:** 1 College of Science and Engineering, Flinders University, Bedford Park, Adelaide, SA 5042, Australia; Kirstin.Ross@flinders.edu.au; 2 College of Humanities Arts and Social Sciences, Flinders University, Bedford Park, Adelaide, SA 5042, Australia; beverley.clarke@flinders.edu.au

**Keywords:** municipal solid waste management, barriers, Thailand

## Abstract

This study focused on determining the barriers to effective municipal solid waste management (MSWM) in a rapidly urbanizing area in Thailand. The Tha Khon Yang Subdistrict Municipality is a representative example of many local governments in Thailand that have been facing MSWM issues. In-depth interviews with individuals and focus groups were conducted with key informants including the municipality staff, residents, and external organizations. The major influences affecting waste management were categorized into six areas: social-cultural, technical, financial, organizational, and legal-political barriers and population growth. SWOT analysis shows both internal and external factors are playing a role in MSWM: There is good policy and a reasonably sufficient budget. However, there is insufficient infrastructure, weak strategic planning, registration, staff capacity, information systems, engagement with programs; and unorganized waste management and fee collection systems. The location of flood prone areas has impacted on location and operation of landfill sites. There is also poor communication between the municipality and residents and a lack of participation in waste separation programs. However, external support from government and the nearby university could provide opportunities to improve the situation. These findings will help inform municipal decision makers, leading to better municipal solid waste management in newly urbanized areas.

## 1. Introduction

Municipal Solid Waste Management (MSWM) refers to waste in a solid form, produced in daily life from households and non-hazardous solid waste from commercial, industrial, and institutional establishments including hospitals, markets, yard and street sweeping [1,2]. Globally, the amount of solid waste is increasing due to population expansion, continuous economic growth [3,4], urbanization and industrialization [5]. In developing countries, high population growth and urbanization, together with rapid economic growth accelerates consumption rates [6]. These patterns have increased the generation rate of municipal solid waste and changed the composition of waste [7]. It is becoming a burgeoning problem for national and local governments to ensure effective and sustainable management of waste. In rapidly urbanizing cities, local governments need to consider the key activities of MSWM including; waste generation and separation, appropriate solutions for recycling, collection, transfer and transport, treatment and proper final disposal [2,3,8]. Inadequate MSWM processes can lead to impacts on human health, living resources and the environment, including water contamination, rodents and insect attraction and flooding due to blocked drainage [3,9,10,11,12,13,14]. Impacts on human health include infection transmission, physical injury, non-communicable diseases, and emotional and psychological effects. In particular, pollutants from landfill can increase the risk of cancer, birth defects, reproductive disorders and respiratory diseases [15]. In addition, inadequate solid waste management systems substantially increase management and disposal costs [4,16]. 

Many municipalities in low and middle-income countries use integrated solid waste management (ISWM) as the ideal principal concept for their MSWM [16,17,18,19,20]. However, different regions have different conditions that require them to determine the best ISWM approach for their situation. ISWM has been described as the integration of sustainable management options, for example: waste minimization, recycling, composting and other recovery options [19,21].

In Thailand, for many years solid waste management has been a topic of heated debate. Solid waste management presents practical management challenges for local government. The need for better waste management has become increasingly obvious with the rise in population. Many potentially good MSWM solutions have been suggested and applied, even if temporarily, in many areas around Thailand, such as waste sorting and recycling [22,23,24,25,26]. However, whilst some have met with success, most have not achieved their objectives. 

Tha Khon Yang, a sub-district of Kantharawichai, Maha Sarakham Province, in northeast Thailand is rapidly changing from a rural to an urban culture. This change has been encouraged partly by the presence of the Mahasarakham University which has a large and rapidly expanding staff and student body [27,28]. Urbanization has resulted in increased waste production, due to new commercial enterprises, accommodation and restaurants. For more than two decades, MSWM has been a serious problem for Tha Khon Yang and the problem is escalating. Between 2009 and 2017 the volume of waste generated in Tha Khon Yang was estimated to double from 4204 tonnes/year to 8004 tonnes per year [29]. 

In 1999, the Thai government formally decentralized many functions from central to local government [30]. Consequently, the Tha Khon Yang Subdistrict Municipality (TKYSM) has primary responsibility for MSWM within its governed service area as identified in the Public Cleaning Act B.E.2535 (A.D.1992) and the Public Health Act B.E.2535 (A.C.1992) [30,31]. The TKYSM receives revenue from the central government via the Provincial authority, and manages waste through its Division of Public Health and Environment. The MSWM system includes collection of waste from stationary waste bins by trucks via a municipal ‘kerb-side’ system that then deliver their waste to a landfill site [29,32]. In Tha Khon Yang there is only limited recycling of materials that hold value (glass, paper, plastic and steel) and this activity is separate from the government’s MSWM system [33].

Residents place their waste bins or plastic bags (of mixed waste) in front of their houses or at unfixed waste collection points along main roads. There are three waste collection trucks in TKYSM that follow a route covering seven waste collection zones [29,32] twice daily from Monday to Saturday (4:00 a.m. to 8:00 a.m. and 1:30 p.m. to 4:30 p.m.). Each truck employs three staff; a driver and two waste collectors who lift waste bins and bags and empty them into the trucks [33].

Around 10 tonnes of waste per day is transferred directly from Tha Khon Yang to a landfill site 25 km away in the same Province. TKYSM pays a disposal fee of 400 baht (US$11.60) per ton of waste to the Province [33]. 

At the time of this study the Thai Government released a national waste initiative that included a ‘No open dumping’ clause [34]. This clause resulted in the closure of the Maha Sarakham landfill site in August 2015. The landfill refused to accept waste from 14 areas outside the Maha Sarakham Municipality [33] and Tha Khon Yang was one of these areas. Tha Khon Yang became very polluted [35] resulting in a state of emergency. Ironically, the closure of the landfill site resulted in the unauthorized dumping of large volumes of roadside waste.

During this time, TKYSM tried to encourage people to manage waste at the source. A public meeting was organized to inform and seek cooperation from the community. However, not many people attended the meeting. In a further bid to inform people about the changed service the TKYSM delivered a message, using a megaphone, from a moving vehicle. This method failed to reach people living in multi-story-complexes or apartments. People were told to manage their household waste by separating recyclables, composting organic waste or, disposing of their waste by burning it. The outcome of the closure and the failed community engagement effort resulted in a dramatic increase of accumulated waste. To reduce waste going to landfill the TKYSM attempted to find space to separate recyclable waste. However, the low-lying topography, lack of private land or civic space for use or purchase has made this a significant and ongoing challenge [33]. 

The theoretical framework applied in this study follows that of the model of Integrated Sustainable Waste Management (ISWM) [36], a model that “allows studies of the complex and multi-dimensional systems in an integral way” [8]. This approach incorporates three key dimensions by which to analyse a waste management system: first; inclusion of the stakeholders who have an interest in solid waste management, second; an understanding of the flow of waste materials from generation points until final disposal, and third; identification/selection of aspects that frame the analysis (such as technical, socio-political, financial aspects). Application of the model has assisted in isolating barriers to effective MSWM in Tha Khon Yang. Clearly defining the barriers may contribute to development of solutions to waste problems both in this region and in other in newly urbanized areas in places suffering similar problems, leading to better MSWM.

## 2. Materials and Methods

To understand the experiences and attitudes of participants with respect to MSWM in the Tha Khon Yang area, and to avoid bias, data was gathered from a variety of sources using a variety of techniques [37] including focus groups, in-depth interviews, observation, and site visits. Research was conducted entirely in the Tha Khon Yang Sub-district. 

This study represents the views of various stakeholders, including both MSWM service providers and users of the service, and some external agents. Stakeholders were chosen according to recommendations from the literature [7,38]. All participants in the study were Thai and over 18 years of age. Service providers were selected from the TKYSM, the local authority providing the waste management service, including directors, official staff and operational staff. The users of the service were people from Tha Khon Yang, including residents (university students, villagers and village leaders), and entrepreneurs (owners or managers of businesses in the immediate area). External agents included other organizations, academics and experts.

The Flinders University Social and Behavioral Research Ethics Committee (Project Number 6784) approved this study on the 21 April 2015. Approval was granted on the English versions of the research instruments. These were later translated into Thai language.

Fieldwork was undertaken between May and September 2015, and July and August 2016. 

Firstly, the primary researcher met informally with the Director and members of the Environmental and Sanitation section of the TKYSM. The Mayor of the municipality was informed about the research project. A formal letter and all related documents were sent to him.

Between May 2015 and August 2016, the primary researcher received invitations to attend six meetings concerning waste management in Tha Khon Yang. These meetings had a number of purposes; some were high-level managers meetings (e.g., with the Provincial Governor, and District Chief Officer) while others were community information sessions. The researcher made audio recordings, took written notes, and observed participants. Attending meetings provided the opportunity to establish rapport with study participants. 

**Figure 1:**
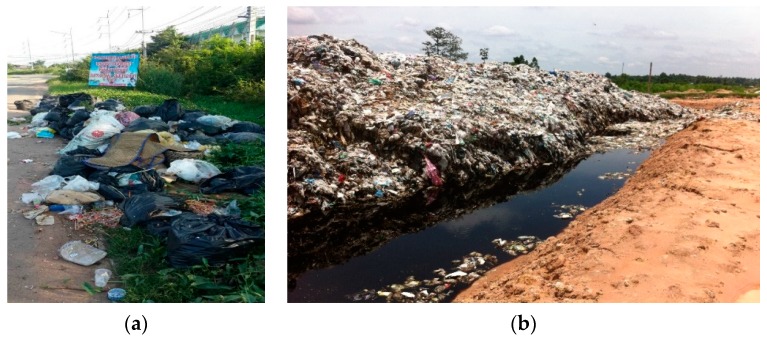
(**a**) Typical scene of road-side waste in Tha Khon Yang; (**b**) Landfill site of Maha Sarakham Municipality.

The primary researcher visited many roads and alleys in villages and communities around the study area to view and take photos of waste piles, bins and waste collecting points. The waste collecting points were also observed, over days, weeks and months. The researcher also visited the landfill site. Figure 1 presents some evidence of pollution. 

Between May 2015 and August 2016, the primary researcher conducted 28 face-to-face, semi-structured interviews with residents, academics, administrators and other organizations related to MSWM of Tha Khon Yang (Table 1). 

Interviews took between 20 and 40 min per person. Times differed between stakeholder groups to accommodate social and cultural backgrounds of interviewees. On deciding to participate, interviewees contacted the researcher and made an appointment. Nonprobability sampling [39] was used for participant selection approaches and techniques that are explained below:

(1) Village leaders: The primary researcher attended a meeting as an observer in Tha Khon Yang community. Attendees of this meeting consisted of the Chief Executive of the TKYSM, leaders of every village in this sub-district (15 villages) and other leaders in this community. The researcher was introduced to attendees at the meeting by the Director of the TKYSM. Volunteer sampling [39] was used and four leaders volunteered to participate.
(2) Entrepreneurs: First, the researcher made an appointment with the Director of the waste management section of the TKYSM who knew the area and its entrepreneurs very well. Then the researcher and the Director selected three groups of entrepreneurs, namely restaurants, dormitories and markets. Purposive sampling [39,40] was used for selecting participants from the list of entrepreneurs in Tha Khon Yang which met the criteria of two urbanized zones and three sizes of businesses (small, medium and large). The researcher visited these entrepreneurs and administered the research instruments.
(3) Academics: The primary researcher sent an invitation to one to two academics from a university and a school ask them to contribute them to participate in the study. The email administered the research instruments. A Snowball technique [39] was used. A key academic referred the primary researcher to other academics who might be willing to participate.
(4) Waste Management Administrators: The primary researcher made appointments with Waste Management Administrators and other external organizations via workplace secretaries. The researcher made a phone call to their secretaries to arrange the administration of the research instruments.

Three focus groups were run, comprising of different stakeholders (waste management operation staff, residents, and students from dormitories in the Tha Khon Yang) (Table 2). The three focus groups were arranged to be held on separate days and in different places, depending on the stakeholder group. Voluntary sampling [39] was used to select local residents from different villages, students (tenants) from different dormitories and waste management operation staff of TKYSM. There were no more than ten participants in each focus group. Focus group sessions lasted for 60 to 90 min. In each focus group session, the research team consisted of the researcher, moderator, note taker, audio recorder and organizer. An audio recording of the focus group conversation was made for later review. During focus group discussions, participants were guided by the moderator who kept the discussion focused, ensured that everyone participated, and encouraged participants to explain their answers. 

The audio files generated from the 28 face-to-face semi-structured interviews and three focus groups were transcribed by the researcher onto a word processor and later uploaded and analysed for thematic content using NVivo software [41]. 

Analysis was undertaken in Thai language to prevent bias or loss of nuance that might arise from translated terms or expressions. Salient quotes were translated into English for use in publication. The analytical framework for this study was constructed of factors or aspects reported in the solid waste management literature and applied by the US EPA (May 2002) [3,42] and Guerrero et al.’s 2013 cross-national study focusing on developing countries [8]. Thematic aspects of the framework include technical, institutional, socio-political, and financial matters.

## 3. Results

The following discussion includes findings from interviews and focus group discussions. The results present the most frequently cited issues. 

### 3.1. Technical and Physical Barriers

A well-functioning waste management system allows residents to dispose of their waste in an appropriate manner. Components of a waste management system include the facilities and equipment used to temporarily store waste (collection bins) or transfer collected waste to its final disposal site [16]. The majority of participants’ comments point to problems with the waste management system in TKYSM. The following sections explain the key technical and physical challenges to MSWM identified by participants.

At least six waste management issues were raised by survey participants that express how current infrastructure is insufficient including; lack of waste collecting points, irregularity of waste collection, inadequate waste collection vehicles, limited access to waste bins, alternative to final waste disposal and improper waste separation facilities. Also physical challenges including; large volume of waste and space limitations.

#### 3.1.1. Lack of Waste Collecting Points

The most frequently cited barrier to effective waste management identified by 15 interviewees (54%) and all three focus groups (100%) was a problem with non-fixed waste collection points. TKYSM does not provide obvious waste collection points for local people. Participants in this study complained that they could not find an appropriate location to put their solid waste, that there were an insufficient number of collection points, or, points are not sited appropriately, or, that collection points are not fixed.

“We don’t know where waste is to be collected from.”

Operational staff of TKYSM F3 [Focus group]

“People do not know where to put garbage. When they see bins or black plastic bags somewhere, they will put their waste there too. The problem is that there is no obvious waste collection point.”

Restaurateur R4 [In-depth interview]

“People who are concerned about waste want to put their garbage in the right place, if there are no waste bin points, they probably cannot do the right thing.”

Leader of village ID01 [Interview]

“Now, the waste collection points have been changed to another area; [collection points] are always changing.”

Market owner M2 [In-depth interview]

Many residents refuse to have a bin in front of their home because they fear others will bring their garbage there too, thereby establishing a neighborhood waste collection point, rather than a household one:

“If we leave bins outside, others throw their garbage here too, for example restaurants and dormitories. It becomes a very untidy place. And sometimes, some pickup trucks bring their waste to this point too… Some waste collecting points are too close to a community or people. That is not very good.”

Restaurateur R5 [In-depth interview]

#### 3.1.2. Irregularity of Waste Collection

Waste collection routes in Tha Khon Yang are divided into seven zones; trucks will collect waste in each zone from Monday to Saturday. Even though there is a system of collection routes for each truck; they struggle to complete their set tasks each day. The waste collectors are unable to adhere to their collection schedule. Waste collection services were a common issue of concern for most participants. Not only residents, but staff at the TKYSM also recognized this problem. The second most commonly cited issue given by 13 interviewees (46%) and three focus groups (100%) was infrequency of waste collection:

“It is an embarrassment, we could not tell [the residents] the exact day of collecting. We have tried to collect every day though.”

Operational staff of TKYSM F3 [Focus group]

“It smells very bad. So I called them (the municipality) to ask when they could collect the waste and how often. They said they were unable to tell me how often that they could offer a waste collection service for this dormitory. If it is a severe problem, I can call them. It is kind of like they will collect randomly. They cannot tell me the exact day.”

Dormitory owner D2 [In-depth interview]

“Oh! When will they [waste collection truck] come? The waste pile is higher than my head already. Sometimes they leave it for 10 days. That is too long. The highest frequency is three collection times a month. Especially these days, there is the huge waste pile because they have not come to collect. We have a trolley to take it away. It takes about 2–3 trips a day to move the waste.”

Dormitory owner D3 [In-depth interview]

“I used to put my garbage bags in front of my restaurant but they weren’t collected. It happened again and again.”

Restauranteur R1 [In-depth interview]

#### 3.1.3. Inadequate Waste Collection Vehicles

The landfill site is about 25 km from the TKYSM and there is no waste transfer station to take waste for sorting. This travelling distance in conjunction with the volume of waste generated each day means that waste collection trucks make a few trips per day to the landfill site. Thirteen interviewees (46%) and three focus groups (100%) stated that deficiencies in waste collection trucks (too few or poorly maintained) were barriers to effective waste management. Participants claimed that each day waste collection trucks are always full before the trucks reach the end of their routes.

“There are not enough trucks or they are out of order.”

Operational staff of TKYSM [Focus group]

“It seems like one waste collection truck could not collect all of the waste from some single dormitories.”

Restauranteur R3 [In-depth interview]

“They [TKYSM staff] said they have only one truck…There was an inadequate number of waste collecting trucks to collect [all of the waste from] the route.”

Dormitory owner D3 [In-depth interview]

“Currently I have heard that the municipality has the budget but they still don’t have ability to buy the truck.”

Dormitory owner D1 [In-depth interview]

#### 3.1.4. Inadequate Access to Waste Bins

In TKYSM waste bins are placed at the kerb-side ready for collection by the waste trucks. Eleven interviewees (39%) and three focus groups (100%) cited inadequate access to these waste bins as one of the main barriers to effective waste management. Waste bins pose a problem for both public and private use. The TKYSM in the past provided kerb-side waste bins for public use in the Tha Khon Yang community but problems developed (for example, some people took the bins away because they were unsightly and smelled bad) and so the TKYSM ceased to provide bins. In the absence of a TKYSM waste bin service most waste bins are now provided by private business (in dormitories) or by households. These private bins are simple receptacles, such as plastic baskets or bins made from old tires. Many people simply use plastic bags. Many respondents mentioned the inadequacy of the capacity of the bins placed in dormitories.

“There are no waste bins [on the kerb-side]. There are only black plastic waste bags. When people put them [on the side of the road], waste becomes scattered [because dogs and scavengers tear open the bags]. Also, in dormitories the [owner] provides one big waste bin on the ground floor. It always overflows; it is not enough.”

Operational staff of TKYSM F3 [Focus group]

“They [the TKYSM] have not provided waste bins for many years. We have to buy them by ourselves.”

Dormitory owner D1 [In-depth interview]

“We separated waste from our room but there is only one bin downstairs…It is a mixed bin and it is collected every day but it is full every day and overflows everyday too.”

F2 Student [Focus group]

#### 3.1.5. Alternatives to Final Waste Disposal (Burning and Illegal Dumping)

When the landfill site limited their intake of waste (on the introduction of the Thai government’s no opening dumping regulations) TKYSM had no option but to reduce its daily waste by more than 10 tonnes. Minimizing waste at the source was the only solution. Residents were required to manage their waste reducing and reusing, as well as disposing of their own waste by burning or burying rubbish on their own land. Four interviewees (14%) talked about alternative methods of final waste disposal, as compared to sending waste to the landfill final. Some TKYSM staff suggested that people dispose of their waste by themselves by open burning or dumping it on their land. However, not everyone can burn or compost their waste (e.g., people living in multi-story complexes or townhouses).

“Sometimes residents want to [compost or burn waste] but the problem is they have no space. It is difficult for them, especially if they live in townhouses.”

Restauranteur R2 [In-depth interview]

“They [the TKYSM staff] suggested that we have to dispose our waste; like burning it ourselves.”

Dormitory owner D4 [In-depth interview]

#### 3.1.6. Improper Waste Separation Facilities

Waste separation is an important strategy to reduce the amount of solid waste going to landfill. It is a goal of the managers of the TKYSM to reduce the amount of waste going to the Maha Sarakham Municipality landfill site because dumping is costly. In addition, the operators of the landfill are limiting the amount of waste they will accept from TKYSM. This means waste separation is an important element of waste management for this local government. However, there is no formal waste separation process in place. Rather, informal systems have emerged:

“They (waste collectors) try to select recyclable waste on the truck too, after our house keepers have already taken some.”

Dormitory owner D3 [In-depth interview]

Participants in this study were cynical about going to the effort of separating their own waste.

“I have noticed the waste collectors throw the waste bags to the truck, then every waste type [recyclables and landfill] are put together anyway.”

Market owner M4 [In-depth interview]

#### 3.1.7. Volume of Waste

Nine interviewees (32%) and three focus groups (100%) stated that the sheer volume of accumulated waste is a primary barrier to effective waste management for the TKYSM.

“Sometimes the waste pile is suddenly high, about my waist, within only a day. I guess it comes from the alleys around here.”

Restauranteur R6 [In-depth interview]

“Now we just collect waste to take it to the disposal site. Sometimes people leave their waste bags after the waste collection truck has already gone (laugh)… There is a lot of waste. Sometimes there is a big pile of waste in front of the dormitory. Each pile is around 2–3 tonnes. We could not collect all of it, we collected it but people dispose of waste again. It’s like a cycle, repeated again and again. It is a lot of waste.”

Operational staff of TKYSM F3 [Focus group]

#### 3.1.8. Space Limitations

Seven interviewees (25%) and one focus group (33%) mentioned the lack of space for waste disposal. The Tha Khon Yang area is flat land with the Chi River running through it. There was a significant flood in 2011 that covered much of the sub-district, indicating that most of the land is not appropriate for a landfill site. Some people said that the limitation of space is an obstacle for managing waste near their residence or place of work.

The TKYSM nominated public places such as the small forest close to the Chi River, a larger forest in the Tha Khon Yang region, and public open spaces as possible sites for a transfer station for waste sorting prior to final disposal. However, the public rejected these suggestions.

“We worked very hard to find a place for a yard for a recycling program. We wanted to use the public space for this project and we organized public hearings many times but people refused it.”

Administration staff ID06 [In-depth interview]

Until recently, Tha Khon Yang was a rural area comprising several small villages. Now it has an increased population density and several main roads connecting a web of smaller roads and alleys. It can be difficult for waste collection trucks to collect waste due to growing traffic congestion. In addition, some of the alleys are inaccessible to the trucks. Therefore, trucks collect rubbish only from the main or easily accessed roads. This problem contributes to the accumulation of waste. 

One interviewee (4%) and one focus group (33%) mentioned the inability of trucks to easily access collection points due to poor condition of roads, limited access to narrow sites such as alleys, and traffic congestion.

“There are dormitories located in small alleys. We have tried to collect the waste there, however the truck could not get in. That causes the [waste accumulation] problem.”

Administrator staff ID05 [In-depth interview]

“Traffic is the obstacle; [the waste collectors] went [to collect waste] in the morning. [This makes it hard for the waste trucks to stop to collect kerb-side waste]. There is a lot of traffic. This is the same every day. Small alleys are especially difficult, because they are narrow.”

Operational staff of TKYSM F3 [Focus group]

### 3.2. Organizational Barriers

Many participants indicated that organizational barriers stand in the way of effective waste management. Five key organizational barriers to effective MSWM in TKYSM include problems for the local authority such as lack of planning, strategic direction, and management (including lack of training) and poor communication between TKYSM staff and the community.

#### 3.2.1. Lack of Planning and Strategy

Planning is normally the first step for designing or developing MSWM [3]. Many participants mentioned poor planning when they talked about challenges to waste management in TKYSM. The director of TKYSM knows that MSWM is a challenge for this area. TKYSM tries to follow the plan from the Maha Sarakham (Provincial) Administrative Organization, however some experts, academics and entrepreneurs suggest that good planning and a strategy for MSWM is absent in TKY. Eleven interviewees (39%) and one focus group (33%) mentioned “lack of planning and strategy”.

*“The policy [of waste management] needs to be clear and earnest, and immediately able to be applied. Action [from the] top down, [and] from the bottom up. Why doesn’t the operator [management of waste in TKYSM] think about this and implement the cycle (of MSWM) from the beginning?”*

Academic ID13 [In-depth interview]

“Here, there is no [waste] management. The municipality needs a new vision for waste management. For example, waste as energy, waste is a resource. The municipality must think outside of the box. They could build a biogas plant from the organic waste. I asked the administrator ‘why don’t you do it?’”

External organization ID12 [In-depth interview]

#### 3.2.2. Inadequate Policy

Respondents at the highest levels from within the TKYSM and business people identified policy inadequacy as a challenge:

Four interviewees (14%) but no focus group talked about “inadequate policy”. One of entrepreneurs said that the TKYSM should have an obvious policy of waste management. Another suggested that an appropriate approach to solve the landfill problem was burning waste from the households. Two others talked about the policy of waste management from the entrepreneur’s perspective.

“I heard from a person who attended the waste management meeting that we need to burn rubbish by ourselves.”

Dormitory owner D4 [In-depth interview]

“The TKYSM needs to have policy from the top. Like, we have to do this, we have to do that.”

Market owner M4 [In-depth interview]

“It is difficult [to create waste management systems [in dormitories], some dormitories, managers are employed to look after the dormitories. They think about the benefit only. That’s it.”

Dormitory owner D1 [In-depth interview]

However, three interviewees from the TKYSM (10% of all interviewees) and a focus group (33%) mentioned that the administrators of the TKYSM pay attention to waste problems and claimed that they have good policy for MSWM. In addition, the provincial administrative organization also puts MSWM as a high priority policy.

“Policy is not the problem.”

Official staff of the Municipality ID14 [In-depth interview]

“We (TKYSM) have tried to make a good plan for MSWM and tried to collect waste and invite entrepreneurs for the [MSWM] meeting. So we worked so hard. ”

Administration staff ID06 [In-depth interview]Poor management/lack of leadership

MSWM is a challenge for local governments especially in developing countries. TKYSM is no exception. Many participants made comments about the inability of the local authority to manage the municipal solid waste of TKYSM. Some comments refer to the ability of the Director, and some to administrators or staff of the TKYSM. Some participants explained that the political challenges associated with these roles, or that staff did not understand their duties or that staff were over worked. 

Nine interviewees (32%) and one focus group (33%) mentioned lack of staff capacity and staff numbers as a barrier to MSWM. Lack of staff capacity was relevant to all levels from the performance of senior management down to street workers.

“The staff don’t understand their duties clearly, because they have to respond to so many issues… health, [supervising] public health volunteers, Health Promotion Hospital and so on.”

External organization ID12 [In-depth interview]

“Our operational staff for waste collection and cleaning need to do other jobs too. Moreover, they are also responsible for managing many cases of waste and wastewater problem. They also need to manage the budget, procure, bla, so many things, but there are only two employees [two official staff of the Division of Public Health and Environment of TKYSM]. As a director, I have to look after all of this. …Inadequate staff and vision of the administrator are the problem. Policy is not the problem.”

Official staff of the Municipality ID14 [In-depth interview]

#### 3.2.3. Lack of Engagement with Programs

To teach people that waste has a value, the TKYSM arranged some practical programs to assist its community to manage MSWM. These programs included earthworm composting and a waste bank (the buying and selling of recyclable waste in schools). However, the programs were introduced only to a few households or schools and are no longer active. When asked about programs for MSWM, TKYSM staff and villagers responded that there were some programs provided by TKYSM but that these were not helpful and that the programs offered were not what was expected. Some residents and the administrator from the TKYSM indicated that there were also some programs designed to develop waste management capacity by taking members of the community to visit and see good practice examples of for waste management facilities in other local organizations. Three interviewees (11%) and all three focus groups (100%) mentioned that the community were disengaged from these programs.

“Yes, yes, we went to Rayong Province and we visited waste projects that cost 20, 200, 300 million [baht]. It is impossible to build those plants in our area. I prefer to see projects in villages that are similar to our villages; projects that could feasibly be applied.”

Residents F1 [Focus group]

“We used to have earthworm compost project in our community. Eventually, villagers didn’t add food waste to the pit but they add some leaves instead. It is incorrect. Then those earthworms die. There are some places that villages still have earthworm projects.”

Administration staff ID05 [In-depth interview]

“The waste bank project in our school has stopped, we could not run it.”

School teacher ID15 [In-depth interview]

#### 3.2.4. Poor Communication

A year prior to the closure of the waste disposal site in a bid to prepare the TKYSM, Maha Sarakham Municipality announced that closure could happen at any time. When the landfill site did close the TKYSM tried to inform its community about the repercussions. However, disseminating information to the urban community in Tha Khon Yang using outdated methods such as inviting people to public meetings or transmitting messages by megaphone from a moving vehicle were not successful. 

Poor communication was the most commonly cited problem raised by entrepreneurs. There were two major issues within poor communication, which are as follows:

• Lack of information

Eleven interviewees (40%) and three focus groups (100%) mentioned that “lack of information” is an issue for effective MSWM for this municipality.

“We need to have an explanation, we need some information to inform us how to discard waste and how can we manage waste.”

Restaurateur R1 [In-depth interview]

• Inappropriate media

Nine interviewees (32%) and one focus group (33%) mentioned that “inappropriate media” was used to inform residents about how to manage their waste in TKY. The information dissemination methods proved ineffective in reaching households and residents.

“I heard that [the announcement] but I didn’t understand what they said. Because it was the announcement by the car and it was driving past… The announcement might not be heard. They only came one time, and so quick…What are they talking about? About the waste, maybe”.

Restaurateur R4 [In-depth interview]

### 3.3. Social-Cultural Barriers

Community participation and awareness are linked directly to MSWM problems. The literature suggests that encouraging people to participate will increase awareness, input and reception [3]. Here, socio-cultural barriers—those social and cultural factors that determine people’s activities—refer to lack of participation, poor co-operation and negative attitudes of residents.

#### 3.3.1. Lack of Participation

Participants in this study claimed that the community was not disposing of rubbish appropriately (e.g., failing to separate waste) and failed to engage in government initiated special meetings designed to instruct people how to manage their own waste to reduce the amount taken to landfill. Participants also claimed that the public ignore instructive kerb-side signs evidenced by the wide-spread practice of rubbish dumping. Respondents claimed this lack of engagement is a barrier to waste management in the TKYSM.

• Lack of engagement with waste separation activities

Seventeen interviewees (61%) and all three focus groups (100%) mentioned a lack of engagement with waste separating activities.

“[Everybody] including students in the dormitories- they do not separate waste. They just throw garbage away.”

Restaurateur R3 [In-depth interview]

“I used to provide separate bins for students [tenants] but they did not separate their waste. Now housekeepers [in the dormitories] separate the waste.”

Dormitory owner D1 [In-depth interview]

• Lack of attendance at community meetings

The TKYSM ran a series of meetings for different villages on the topic of managing domestic waste. Six interviewees (21%) and two focus groups (67%) talked about people not joining these TKYSM meetings. Many entrepreneurs did not participate in the meetings for different reasons such as being “*too busy*” or considering that “*it is useless to attend*” or for “*no reason*”.

“When the municipality invited us for the meeting, we could not go because it was scheduled for 9 a.m. Many shop owners and employees could not go. At that time, every shop is busy and we have to get our shops ready to open.”

Restaurateur R6 [In-depth interview]

“I didn’t go [to the meeting], let them [the TKYSM staff] think by themselves. Even if we attend the meeting, they won’t follow our suggestions. They [the TKYSM staff] just need us to attend. They will do what they want anyway.”

Dormitory owner D1 [In-depth interview]

• Failing to observe signs

Two interviewees (7%) and a focus group (33%) suggested that many people in the community ignore [locally constructed] signs instructing, “do not litter” or “do not put your garbage here”.

“Lack of participation of entrepreneurs is a significant problem: restaurants, dormitories, especially dormitories. This morning I have just put up the sign [‘Please do not litter in this area’]. I also cleaned the scattered waste around the kerbside not far from my restaurant] after the waste collection truck had collected. Then, by late morning, there were some people who had put the waste there [the same place]. Also some waste pickers had scattered the waste there [the same place] again.”

Restaurateur R4 [In-depth interview]

#### 3.3.2. Lack of Co-Operation

Some participants talked about the conflict between the TKYSM and the Mahasarakham University and who should take responsibility for waste collection along the main roads leading to the University. Some participants mentioned a lack of participation by private businesses. Twelve interviewees (43%) and three focus groups (100%) mentioned poor cooperation.

“The cause of problem is that there is a conflict between the university and this municipality, which is a barrier. If the municipality [TKYSM] cooperated with the university, this problem would have been solved a long time ago…there is a public area, but the municipality said it belongs to the University. But the municipality has the responsibility to look after this area. So if the municipality [TKYSM] doesn’t want to do it, they have to transfer this authority to the University.”

Dormitory owner D1 [In-depth interview]

Some mentioned poor cooperation between dormitory owners and residents.

“We provided the separate waste bins but they are useless, residents don’t separate their waste; all the bins are used for mixed waste. So it doesn’t matter which color they are.”

Dormitory owner D2 [In-depth interview]

#### 3.3.3. Negative Attitudes

Participants mentioned that a barrier to MSWM in the Tha Khon Yang area was related to the negative attitudes of people including residents (and students), entrepreneurs and local municipality staff. Lack of concern; blaming others; and believing waste management is unsolvable are examples.

• Lack of concern for waste management

Answers given by study participants illustrate a lack of understanding of how individuals contribute to MSWM. Common perceptions were: that business owners do not contribute a significant amount of waste (18%), that people don’t care about pollution (14%) and that people have no time to manage waste (11%).

“Even through people were educated about waste management they were not concerned about it. That is because the habit of Thai people is just ‘take it easy’. Most Thai people are like that. We do not exactly love our environment, I think. Unlike some countries that have experienced a disaster, like Japan, for example. They are really concerned about their environment.”

Market owner M1 [In-depth interview]

“I cannot do that [dispose of my waste thoughtfully], I do not have time. And there is not a lot of sellable waste. For others [waste pickers], that is their job, let them have their job.”

Market owner M2 [In-depth interview]

• Blaming others

Eleven interviewees (39%) and two focus groups (67%) blamed others for bad behavior with respect to waste management. In response to the question about who should be responsible for making the changes for the better MSWM some participants replied as follows:

“Our waste is dry waste. Nowadays it smells because of waste from others, for instance the noodle shop, they will throw their waste here around 1 am. The smell is not because of our market waste.”

Market owner M2 [In-depth interview]

“It is because the big dormitories, they produce a lot of waste. There is not a lot of waste generated from our village.”

Restaurateur R3 [In-depth interview]

• Insolubility of the problem

Seven interviewees (25%) indicated that they thought that the waste problem is too difficult to solve because it is not possible to change the behavior of people.

“Waste producing behavior cannot be changed. This is Thailand. We always discard waste. But if it is about waste separation, we can do that.”

Market owner M4 [In-depth interview]

“It is difficult to change children’s behavior. They have no discipline. For example, if there is a bin, they will throw their rubbish to the side, not in the bin. This is the habit of children now-a-days.”

Teacher ID15 [In-depth interview]

• Insufficient communication

Nine interviewees (32%) mentioned communication problems between entrepreneurs and their staff or between tenants and owners of dormitories. If entrepreneurs do not set rules or provide guidance for staff, it is difficult to control the waste management practice in these places. Communication problems emerged between the restaurateurs who generate organic waste and farmers who want to use it. There were allegedly agreements made between some restaurants and farmers but farmers were reneging on their deal.

“Many people used to ask me for my food waste [for example, farmers who would feed their livestock]. But they didn’t come to get it.”

Restaurateur R1 [In-depth interview]

“Last year the town [Maha Sarakham] municipality stated that the waste management system has to be prepared because the disposal landfill will be closed whenever. That was a warning. About a month before the landfill was closed, the TKYSM was informed about the closure again. But the TKYSM didn’t tell any [local] entrepreneurs. We got a letter about one day before the landfill was closed. We have to manage our waste by ourselves, they won’t collect the waste. And the day after, 12 August they [TKYSM] distributed an invitation letter at 5:00 a.m. to attend a meeting about waste at 9:00 a.m. the same day. I don’t know how many people attended the meeting. I didn’t go.”

Dormitory owner D1 [In-depth interview]

“The staff usually peeling the fruits here and they didn’t care. But I cannot do anything about this sometimes, I tried to tell them. Sometimes, we need to let it be.”

Restauranteur R2 [In-depth interview]

“If I tell them [tenants] to [separate waste], will do it or not? Hmm… I am not sure. (Laugh)”

Dormitory owner D2 [In-depth interview]

### 3.4. Financial Barriers

To create a MSWM system it is necessary to consider financial factors. This refers to waste fees, including the public’s ability and willingness to pay and the ability for the collection of fees by the municipality; and it also refers to the public’s attitude to the value of waste. The majority of participants indicated that financial constraints are one of the major barriers to effective solid waste management of TKYSM. There are three main financial barriers; waste management fees; the assumption that waste has no value; and that overall, there is insufficient external funding. 

#### 3.4.1. Waste Management Fee Collection

TKYSM does not have a clear fee system for waste collection services. Normally, commercial businesses pay a fee (tariff) at the TKYSM office annually or monthly with different rates that depend on the type of business. Every household pays a tariff to the TKYSM of 10 to 30 baht (US$0.29 to US$0.87) per month (depending on the size of the household). In this study fees were the most commonly cited financial issue. Nine interviewees (32%) and one focus group (33%) stated that fee collection was a barrier for effective MSWM. Most participants said that they were willing to pay the waste collection service fee and that the price of the fee was reasonable. However, some people refuse to pay the waste management service fee because they claim to manage their own waste and do not use the service provided by the TKYSM. Alternatively, some restaurants and dormitories paid additional fees to a private waste collector. These respondents said they gave extra money (300–1000 baht (US$8–$28), per month) to waste collectors to clear waste from their dormitories.

*"In the past the authority [*TKYSM*] collected 50 baht [waste collection fee], I think…They [the authority from* TKYSM*] hardly come now, it’s hard to get the authority to come. Some authorities collected the fees and did not hand them to the council. I heard that sometimes no authority collect fee! …Some people throw their waste in the field or into someone else’s property. Some people have different thinking, they are afraid to pay so they decide to throw the waste elsewhere."*

Restauranteur R3 [In-depth interview]

“I give them [waste collectors] 500 baht a month and give them beers [personally] plus the tips…If you charge per unit, 5 baht per unit, and 10 units. We [normally] pay 50 baht per month [to the TKYSM]. I was asked why I paid that much? Some big apartments only paid 30–40 baht; I don’t know why, I just gave…The fee collector has changed several times. They’re scared to come now. ‘They’re afraid they will get scolded.”

Dormitory owner D5 [In-depth interview]

“We had paid 10 baht per month, no problem. And we paid for the bins that are alright… But, some families have said that they didn’t use the waste collection service; so they do not pay the fees as it is not worth it… It’s okay if the authority was collecting for the fees.”

Residents F1 [Focus group]

For this issue, the administrator of the TKYSM has said that the normal fee rate is reasonable. *“The rate of waste collection fee is normal, so people did not suffer, just 10 baht per household.”*

Administration staff ID05 [In-depth interview]

#### 3.4.2. Insufficient Funding

TKYSM currently pays for the rubbish trucks, maintenance, fuel, collection, and waste disposal. Individuals are responsible for providing their own bins. Six interviewees (21%) mentioned that insufficient funding is a barrier to the effective waste management for the TKYSM. Some residents responded that they thought that the TKYSM might not have adequate funds for effective waste management. However, the TKYSM indicated that there was enough funding for MSWM. Funds come from the annual payments for waste management from the central Thai government, together with the fees paid by residents and businesses. In addition, the Department of the National Resources and Environment Office of Maha Sarakham, can provide further funding if requested.

“There might be limitations on the budget [of the TKYSM]. The large number of nonregistered population [not being included in the budget]; not enough money. It is because the expected volume of waste [that is calculated] is not covered by the budget.”

Dormitory owner D4 [In-depth interview]

“It is difficult to encourage villagers to participate. We [waste administration of the TKYSM] have arranged waste projects every year, but it seems like we wasted money.”

Administration staff ID05 [In-depth interview]

Two staff from TKYSM (67%) claimed the budget was sufficient for MSWM:

“We have enough money [for MSWM].”

Administration staff ID06 [In-depth interview]

A number of business people indicated they have considered providing different types of bins for their apartments. However, they have not yet because they are waiting for [general] local economic improvement.

“The business is not good so this [changing the bins] cannot be done now because the economy is terrible. We want to do it [change the bins’ color]; but we need to wait for the right time.”

Restauranteur R2 [In-depth interview]

#### 3.4.3. Waste Has No Value

Waste is valuable if there is a demand for discarded materials. Waste separation is the first step. For example, waste is of value to waste pickers who sell recyclable materials, and to farmers, who can turn organic waste into compost or feed for their stock. However, some participants from commercial businesses do not separate waste because it has no value to them, and waste separation takes time. Five interviewees (18%) suggested that “waste has no value”. Such a perspective is another barrier to the effective waste management for the TKYSM.

"I do not have time to collect it [recyclable waste], I rarely do it. There is not much to collect. For others [waste scavengers], that is their job; they have to make a living."

Market owner M2 [In-depth interview]

"The value [of recyclable waste] is little. It could be 30 baht to 40 baht, it is cheap. Five bottles, two baht per a kilo. They are light and it takes a long time to collect. There are not many of them, so I let the housekeeper take them. I don’t want them."

Dormitory owner D3 [In-depth interview]

### 3.5. Legal and Political Barriers

Many participants indicated there were legal-political barriers to effective solid waste management. Inadequate and weak legislation and conflicting interests were cited as such barriers.

#### 3.5.1. Inadequate and Weak Legislation

Four interviewees (14%) and a focus group (33%) mentioned that “inadequate legislation” was a barrier to effective waste management for the TKYSM. One entrepreneur noted that there are no rules for waste management in some dormitories. Another mentioned the weakness of the regulation. Some villagers highlighted that because punishments for dumping rubbish are inadequate or weak, this behavior continues.

“The TKYSM needs to have rules [about waste management], more obvious rules to guide the residents. What do they want us to do? We need to help each other. They need to set the rules and we must follow the rules.”

Market owner M3 [In-depth interview]

“For example, even they [the TKYSM] have the rules, however people still do the same [wrong thing]. People are not afraid to be fined…’If you discard the garbage here, you will be fined for 500 baht’; nobody follows that because the writer just wrote the sign but has never fined anyone. Don’t be afraid, you will never be fined.”

Restauranteur R5 [In-depth interview]

#### 3.5.2. Conflicting Interests

Interviewees noted that political problems could be as barriers to effective waste management. Individuals elect to fill administrator positions in local organizations and residents vote. Four interviewees (14%) but no focus group suggested that the elected administrator in order to protect their position avoid conflict. This holds true for politicians too. Respondents thought politicians were afraid to make decisions about waste disposal sites that were unfavorable with residents or the community because they will not be re-elected.

### 3.6. Population Growth

With the increasing size of the TKYSM population there is a concomitant increase in the amount of waste. There are permanent and non-permanent residents complicating this matter. The budget TKYSM receives from the Thai central government covers only permanent residents. This anomaly was noted and that TKYSM’s budget needs to be supplemented for non-permanent residents who put pressure on the existing system. Nine interviewees (32%) and a focus group (33%) talked about “population growth” as a barrier to effective waste management for the TKYSM.

“There were too many non-registration populations (temporary population), so the budget would not be enough. Also the amount of waste would not be calculated correctly.”

Dormitory owner D4 [In-depth interview]

## 4. Discussion and Recommendations

The in-depth interviews, focus groups, observations and secondary data, all point to participants not being satisfied with the waste management service from the TKYSM. A SWOT analysis of the results, highlight two main factors for the TKYSM to consider, internal factors and external factors [43,44]. Table 3 presents an overview of the SWOT analysis.

### 4.1. Internal Factors

Internal factors including technical aspects of MSWM, organizational aspects (staff, policies, plans and strategies), and funding relating to what is happening within the TKYSM.

#### 4.1.1. Insufficient Waste Management Infrastructures

The first internal barrier to overcome is the ineffective MSWM system, the service provided by TKYSM including the waste collection, transfer and disposal.

Infrastructure for of waste operations is lacking (not enough waste bins, waste collection services, and waste collection vehicles). No separation bins are provided; people leave waste in plastic bags beside roads. Some households purchased waste bins but then others used these bins too. People do not know where to put their garbage because there are no fixed waste collection points or times for garbage collection. The TKYSM tries to solve the problem on a day-to-day basis. The waste collection staff collect waste every day but there is still an accumulation of waste. This causes pollution and nuisances such as odor, an increase in disease vectors (flies and dogs) and unsightliness. While the TKYSM has a plan for waste collection routes; waste collectors or staff do not follow the plan because the trucks are usually full before the end of the routes, or the trucks cannot enter some of the route. 

#### 4.1.2. Organizational Barriers

Opportunities for improvement in organizational structure to create more effective MSWM are discussed below.

#### 4.1.3. Communication

The TKYSM needs communicate more effectively by providing clear information to people encouraging them to take some responsibility for waste management in their households and businesses. Additionally, people need to understand that actions of individuals influence how the system works. TKYSM should communicate using methods that will reach different groups in the community. 

#### 4.1.4. Staff

The directors of the TKYSM have policies to support better MSWM. However, strategies to put these policies into action are weak. It is important for organizations to work together to improve implementation of strategies. Staff numbers and capacity within the TKYSM is a weakness. MSWM is but one responsibility in the sanitation and environmental section of the Public Health Division of TKYSM. There are only two official staff with too many responsibilities; their roles also cover environmental sanitation, health promotion, and occupational health. They fulfill roles of sanitation inspector, public awareness trainer, they attend to administrative tasks in the office and do fieldwork [45]. Employees have insufficient education to conduct their work. The operational staff are also required to respond when other sections of the TKYSM call on their assistance. 

#### 4.1.5. Information

It is difficult to make reliable decisions on an appropriate waste management system without appropriate information. There is little data collected. Details of waste collection was being recorded in handwritten notebooks that are not considered to be of high importance. For example, when asked for these records, staff could not find one of these notebooks. There are other missing records, including historical records and reports. There is limited data upon which to base decision-making. It is not possible currently to identify trends in change of volume of waste, seasonal variations, type of waste and so on. There is no database to inform managers. Related databases are scattered across institutions that have conducted relevant studies, such as Mahasarakham University and Maha Sarakham Town Municipality, suggesting that a cooperative strategy to collate this data would be successful.

#### 4.1.6. Financial Barriers

Finance is an important issue for MSWM [3]. It was found that financial problems were common among waste processing facilities, including imbalances between revenue and expenditure [16]. Like many Asian cities, TKYSM MSWM budget is spent on collection and disposal rather than supporting waste minimization at the source [46]. Troschinetz and Mihelcic found that finance is one of the three biggest barriers for developing a recycling system in developing countries; on the other hand, household economics is one of the smallest barriers [47]. 

Many residents are willing to pay for waste collection. Some people said that they would still be willing to pay more if the service improved. TKYSM has a problem with irregularity of collecting fees complicated by variation in price charged to different users of the service. The TKYSM needs to develop an appropriate rate and a payment system that is convenient for residents. 

Notably, the staff of TKYSM said that they think that there is enough money for waste management. However if the MSWM system is to be improved, staff consider that waste management demand more of budget. Funds allocated by the Thai government could be used to undertake training projects within the community. However, accessing these funds requires staff capacity to draft and submit and successful proposals. The problem is reflected elsewhere; Kotuta and Sobhanaboon found that lack of staff capacity in Maha Sarakham Municipality is a cause of waste management and waste collection problems. They also reported that a fee system needs to be developed there as well [48].

### 4.2. External Factors

External factors—those factors not in the control of the TKYSM but that affect is MSWM include social and public participation and cooperation from other related organizations and policy, legal, political barriers, and physical barriers are included. 

#### 4.2.1. Social-Cultural Barriers

Negative attitudes and behavior of residents could be seen as social and cultural barriers; limited waste separation by residents, students and entrepreneurs is a barrier to effective waste management. This is a difficult problem because it is only partly controlled by the TKYSM, and is compounded by the rise in population, including large numbers of temporary residents. 

In general, people think the value of recyclable waste is very low, so there insufficient incentive to separate waste materials. Business people had varied ideas about the value of waste. Some suggested that while waste might have value, they still see collection and reuse of waste as not worth doing. Mostly, they leave the recyclable waste to housekeepers to collect. The waste then becomes worth money to those who collect and/or sell the waste. However, most people think that is the quick and easy solution for taking their waste away. 

Implementing successful recycling and composting programs is important work for the local government. Getting people to consider the value of waste and think before throwing things away can significantly reduce waste volume. “Valorisation” of materials is the basis for all private-sector recycling activity, meaning that even if the owner of an item throws it away, it still has some retained value [7]. Separating waste into recyclable waste, organic waste, and general waste has been successful elsewhere [7,16,23]. Composting particularly is likely to be a good investment in Tha Khon Yang because there is much agricultural land. This would require building better facilities to compost organic waste. However, researchers have indicated that farmers in Maha Sarakham were concerned about inadequate nutrient in organic compost [49].

“*It is hard to solve the problem*” was a common sentiment. “*This is the habit of Thai, it is impossible to change*”. Addressing these attitudes requires input from many sections and the TKYSM needs to organize training or education programs to encourage people to be concerned about their waste problems and to change their habits, particularly with respect to waste separation. Promotion of public participation or education campaigns is needed to encourage residents to separate waste at source and to increase the recycling rates [7]. TKYSM needs to understand their residents’ culture and determine suitable solutions to develop a new system that covers the whole process of waste management. 

There is a lack of communication between local government and public. Provision of information about waste management is insufficient and the format of disseminating information needs modification so that people can understand. Lack of awareness of the population was a concern for the entrepreneurs. However, based on the students (tenants) and other residents’ focus group, it seems there is awareness within some groups about the value of waste separation. These groups indicated that they are ready to separate waste if the separating waste system is easy to understand and follow. From observations made during the community meetings, the researcher witnessed that villages leaders are both willing to participate and are very concerned about waste management.

#### 4.2.2. Legal and Political Barriers

There are good policies for MSWM in TKYSM but poor strategies to solve waste problems in the area. This makes it difficult for people to understand how to participate or follow the policies of the TKYSM. For example, from the attitude of participants it was clear that consideration of waste management by businesses is still poor; many businesses have not developed or implemented waste management systems. 

The TKYSM claimed they had tried to encourage people to separate waste; however they failed to enforce this. This suggests that legal implementation is required. Tightening of laws may encourage waste separation, and the TKYSM should establish their own rules to support their system and encourage people to participate in the waste management system. 

The political context of local government affects MSWM in many ways [2]. Because the Director of every municipality is elected by the public, there is pressure on the Director to make popular decisions that will help them retain their role for as long as possible. This makes the implementation of change that will affect peoples’ lives a challenge, and encourages a status quo. 

#### 4.2.3. Physical Barriers

Tha Khon Yang area is flat land and flooding is common. It is difficult to find suitable space for waste recycling and disposal both because of the geography and opposition from local people who are afraid of pollution and who do not trust that waste will be managed correctly. This lack of space is identified in the waste management and hazardous waste plan for the Maha Sarakham Province (2015–2019); and the requirement for large areas of land for new waste disposal in this area [50]. 

In addition, Malaysia [51] and Vietnam [52] are experiencing population increases in immigration and large numbers of unregistered population, and an increase of visitors places like in Bang Saen, popular beach in Thailand [53] or in Bali, Indonesia [54], making it difficult to create waste management plans. 

Principally, planning is the first step in designing or improving a waste management system [3]. Proper waste collection is an essential function of government authorities. Waste collection is one of the key components of an integrated sustainable waste management system and it is one of the main functions of urban services [7,55]. Therefore, the TKYSM needs to overhaul its waste collection system with a clear plan for MSWM including minimizing waste generation, more efficient and effective waste collection, transfer, and transportation to a final disposal site. With the centrally managed budget, the TKYSM needs to have a plan to spend money on waste management for the long term. Purchase of additional trucks was a common suggestion from residents; however, this option will be very costly. Redesigning waste collection routes with increased frequency of waste collection or waste transfer points and a waste separation system, together with residential or waste separation at source, might mean that the additional truck purchase is not necessary in the short term. More immediately, the TKYSM needs to prepare an emergency plan for situations such as when the landfill disposal site closes. Currently, methods used to deliver information are inappropriate or ineffective. Using appropriate technology (e.g., creating brochures, use of internet, emails, etc.) to improve information transfer will require training and human resource development.

## 5. Conclusions

This study has found the waste management system of the Tha Khon Yang Subdistrict Municipality cannot support the current increase in waste generation. Opinions from various stakeholders provided significant insight for the TKYSM to consider. There are many barriers that the TKYSM must overcome including: technical, organizational, social-cultural, financial, legal-political and population growth. 

The findings of this study will help the TKYSM policy makers develop an effective and appropriate MSWM for Tha Khon Yang. This study may lead the way to develop a new waste management approach and a new waste management system. This new approach could be adapted to other local areas in Thailand or other developing countries that are facing similar problems. 

## Figures and Tables

**Table 1:** In-depth interviews.

In-Depth Interview Participants	Number of Participants
**Residents**	**19**
Leaders of villages	4
Restauranteurs	6
Dormitory owners	5
Minimart Owners	4
**Academics**	**4**
University lectures of Mahasarakham University	3
School teacher of Ban Hua Khua School, Tha Khon Yang Sub-district, Maha Sarakham Province	1
**Administrators**	**3**
Administrators of Tha Khon Yang Subdistrict Municipality	
**Others organization that related to municipal solid waste management**	**2**
Director of the Provincial Natural Resources and Environment Office, Maha Sarakham Province Waste operator of Maha Sarakham Town Municipality	
**Total**	**28**

**Table 2:** Timetables for conducting focus groups.

Date	Time	Focus Group Participants	Number of Participants
31 August 2015	(1.30 h)	Waste management operational staff of the TKYSM	10
1 September 2015	(1 h)	Students, living in private dormitories in Tha Khon Yang	6
15 September 2015	(1 h)	Residents living in Tha Khon Yang	8
		**Total**	**24**

**Table 3:** SWOT analysis for MSWM in Tha Khon Yang Subdistrict Municipality.

	Positive	Negative
**Internal**	**Strengths**	**Weakness**
	Good policy Enough budget Location is not too far from the disposal site (25 km approximately)	Weak strategy Weak and inadequate regulation Lack of planning Insufficient waste infrastructures (waste bins, waste collection points, waste infrequency waste collection, waste collection and transfer vehicles) Waste fees collection system Lack of information system and training program Lack of staff capability Staff have too great a workload Lack of systems to ensure that staff are rotated through a range of job roles Political influence at the organization level
**External**	**Opportunities**	**Threats**
	Funding from central government National and provincial levels have policies that support waste management and also be the driver for the TKYSM to develop better MSWM Mahasarakham University could support training or knowledge Peoples’ concern about waste problems	Lack of control over operation of disposal site (the TKYSM did not operate or control the waste disposal site, it is owned and operated by another organization) Flat land and flooding location Poor cooperation from residents (especially entrepreneurs) People are unwilling to separate waste at source Increasing population and economic growth may increase consumption and waste Low value (price) of recyclable waste
